# Social media platforms generate billions of dollars in revenue from U.S. youth: Findings from a simulated revenue model

**DOI:** 10.1371/journal.pone.0295337

**Published:** 2023-12-27

**Authors:** Amanda Raffoul, Zachary J. Ward, Monique Santoso, Jill R. Kavanaugh, S. Bryn Austin

**Affiliations:** 1 Division of Adolescent/Young Adult Medicine, Boston Children’s Hospital, Boston, Massachusetts, United States of America; 2 Department of Pediatrics, Harvard Medical School, Boston, Massachusetts, United States of America; 3 Center for Health Decision Science, Harvard T.H. Chan School of Public Health, Boston, Massachusetts, United States of America; 4 Department of Social and Behavioral Sciences, Harvard T.H. Chan School of Public Health, Boston, Massachusetts, United States of America; University of Pisa, ITALY

## Abstract

Social media platforms are suspected to derive hefty profits from youth users who may be vulnerable to negative mental health outcomes, including depression, anxiety, and eating disorders. Platforms, however, are not required to make these data publicly available, which may limit the abilities of researchers and policymakers to adequately investigate and regulate platform practices. This study aimed to estimate the number of U.S.-based child (0–12 years old) and adolescent (13–17 years old) users and the annual advertising revenue generated from youth across six major platforms. Data were drawn from public survey and market research sources conducted in 2021 and 2022. A simulation analysis was conducted to derive estimates of the number of users and the annual advertising revenue per age group and overall (ages 0–17 years) for 2022. The findings reveal that, across six major social media platforms, the 2022 annual advertising revenue from youth users ages 0–17 years is nearly $11 billion. Approximately 30–40% of the advertising revenue generated from three social media platforms is attributable to young people. Our findings highlight the need for greater transparency from social media platforms as well as regulation of potentially harmful advertising practices that may exploit vulnerable child and adolescent social media users.

## Introduction

Young people are using social media more than ever [1]. One public research study found that, as of 2022, nearly half of adolescents reported being online “almost constantly,” up from 24% in 2015 [2]. A growing body of research documents associations between social media use, social media advertising, and negative mental health outcomes among youth, including depression, anxiety, and eating disorders [3–5].

However, despite the potential risks of social media for youth mental health, under current U.S. law, social media platforms have no legal obligation to release data surrounding the types of content youth are exposed to, nor the impacts of such content [6]. The lack of transparency among social media platform practices and the platforms’ reluctance to share data with researchers and journalists has been well-documented [7–9].

Social media platforms are highly incentivized to keep youth online–children and adolescents’ online experiences are heavily monetized through advertising revenue on platforms’ websites and mobile applications [10, 11]. Platforms draw upon highly personalized computational advertising to match users’ specific demographics and usage patterns with advertisers’ financial interests [12]. Although computational advertising may pose benefits to consumers, there are ethical concerns regarding the amount of data it may require from vulnerable users, such as youth. Since regulatory agencies do not hold social media platforms sufficiently accountable for their engagement with, collection of data for, and effects of advertising on children and adolescents [6], they are not required to report advertising revenue nor the age breakdown of users.

In this study, we obtained data from a business marketing source and from public survey data to conduct a novel simulation analysis to provide the first known estimates of the number of users and the annual advertising revenue generated from U.S.-based child users (ages 0–12 years) and adolescent users (ages 13–17 years) for six major social media platforms (Facebook, Instagram, Snapchat, TikTok, Twitter, and YouTube) in 2022.

## Methods

### Data sources

Age-specific estimates of 2021 population size were obtained from U.S. Census [13]. We obtained estimates of the percentage of the population who are users of each platform by age group from a range of sources. Estimates for children and adolescents in 2021 were obtained from a nationally representative survey conducted by Common Sense Media, a nonprofit research and advocacy organization [1]. Social media was defined in the Common Sense Media survey as including websites and applications such as Snapchat, Instagram, Discord, Reddit, or Facebook; platforms such as YouTube, TikTok, and Twitch were considered online video sites. Sampling error was estimated as 3.2% for the full sample. Estimates for the use of social media among adults in 2021 were obtained from a nationally representative survey of 1,502 U.S. adults conducted by Pew Research [14]. Sampling error (+/- for 95% CI) was estimated as 7.3% for ages 18–29, 5.2% for ages 30–49, and 5.8% for 50–64 and 65+. We have summarized the percentages by age group in S1 Table.

We obtained estimates of projected annual gross advertising revenue in 2022 for each platform from eMarketer (https://www.insiderintelligence.com/) [15], summarized in S2 Table. These data are not publicly accessible without a subscription and were accessed through a Harvard University library subscription. Data were downloaded on April 28, 2022. We also used eMarketer data to estimate the total users of each platform by age group (S3 Table). We obtained estimates of average minutes per day among users by platform for youth (<18 years old) in 2021 from Qustodio [16] and adults (18+) from eMarketer, summarized in S4 Table.

### Analysis

To estimate age-specific revenue for each platform, we modelled the number of users and mean duration of exposure by continuous age, synthesizing data from multiple sources to produce estimates of platform use consistent with survey data and platform-specific estimated revenue. Probabilities of platform use and duration of use (minutes per day) were sampled from estimates by age group, accounting for sampling error, and interpolated using natural cubic splines to produce age-specific estimates. Due to lack of data, we assumed that revenue per minute of platform use was constant by age. Parameters for each platform were fitted separately using a stochastic optimization algorithm, and the 100 best-fitting parameter sets were used to estimate the mean and 95% uncertainty intervals (UI, 2.5 and 97.5 percentiles of simulation results) of age-specific revenue to account for parameter uncertainty. The model was developed in R (v3.6.1).

## Results

### Estimated number of youth users across major social media platforms

For each of the six major social media platforms (i.e., Facebook, Instagram, Snapchat, TikTok, Twitter, and YouTube), we present model estimates for the number of youth users in the U.S. in **Table 1**. In 2022, among children ages 0–12 years and adolescents ages 13–17 years, the most popular platform was YouTube, which had nearly 31.5 and 18.4 million users, respectively, in these age groups. The least popular platforms among youth were Twitter and Facebook, which had approximately 7 million and 9.9 million users ages 0–17 years. Instagram, Snapchat, and TikTok had an average of nearly 18 million users under the age of 18 years.

**Table 1:** Estimated number of U.S.-based youth users of six social media platforms, by age group, 2022.

Social Media Platform	Number of Users by Age Group: Mean (95% UI)		
	Ages 0–12 Years	Ages 13–17 Years	Ages 0–17 Years
Facebook	2,757,000	7,047,000	9,904,000
	(2,825,000–2,892,000)	(7,022,000–7,070,000)	(9,872,000–9,936,000)
Instagram	3,982,000	12,726,000	16,708,000
	(3,922,000–4,037,000)	(12,688,000–12,779,000)	(16,660,000–16,758,000)
Snapchat	2,869,000	15,131,000	18,000,000
	(2,723,000–2,946,000)	(15,066,000–15,253,000)	(17,974,000–18,022,000)
TikTok	3,041,000	15,932,000	18,972,000
	(2,860,000–3,221,000)	(15,753,000–16,078,000)	(18,753,000–19,242,000)
Twitter	2,071,000	4,929,000	7,001,000
	(1,926,000–2,155,000)	(4,849,000–5,082,000)	(6,982,000–7,020,000)
YouTube	31,448,000	18,341,000	49,789,000
	(31,184,000–31,708,000)	(18,070,000–18,596,000)	(49,744,000–49,835,000)

### Estimated advertising revenue generated from youth users across major social media platforms

**Fig 1:**
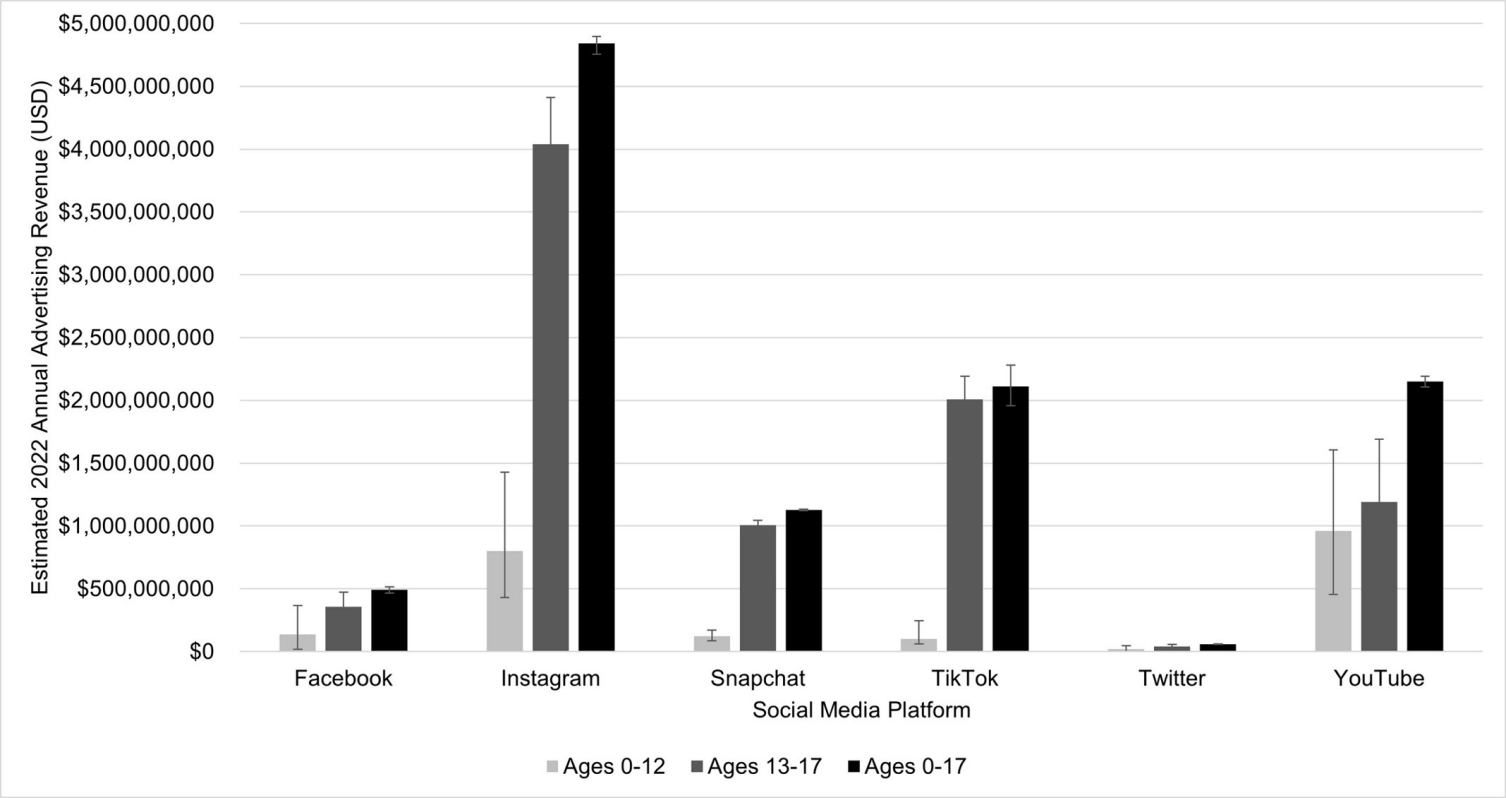
Estimated annual U.S. advertising revenue from users under 18 years of age across social media platforms, 2022.

The age-specific advertising annual revenue derived from youth ages 0–12, 13–17, and 0–17 years from 2022 is presented in **Fig 1**. The greatest advertising revenue profits derived children ages 0–12 years old was from YouTube ($959.1 million), followed by Instagram ($801.1 million) and Facebook ($137.2 million). Among youth ages 13–17 years old, the greatest estimated advertising revenue was generated on Instagram ($4 billion), TikTok ($2 billion), and YouTube ($1.2 billion).

**Fig 2:**
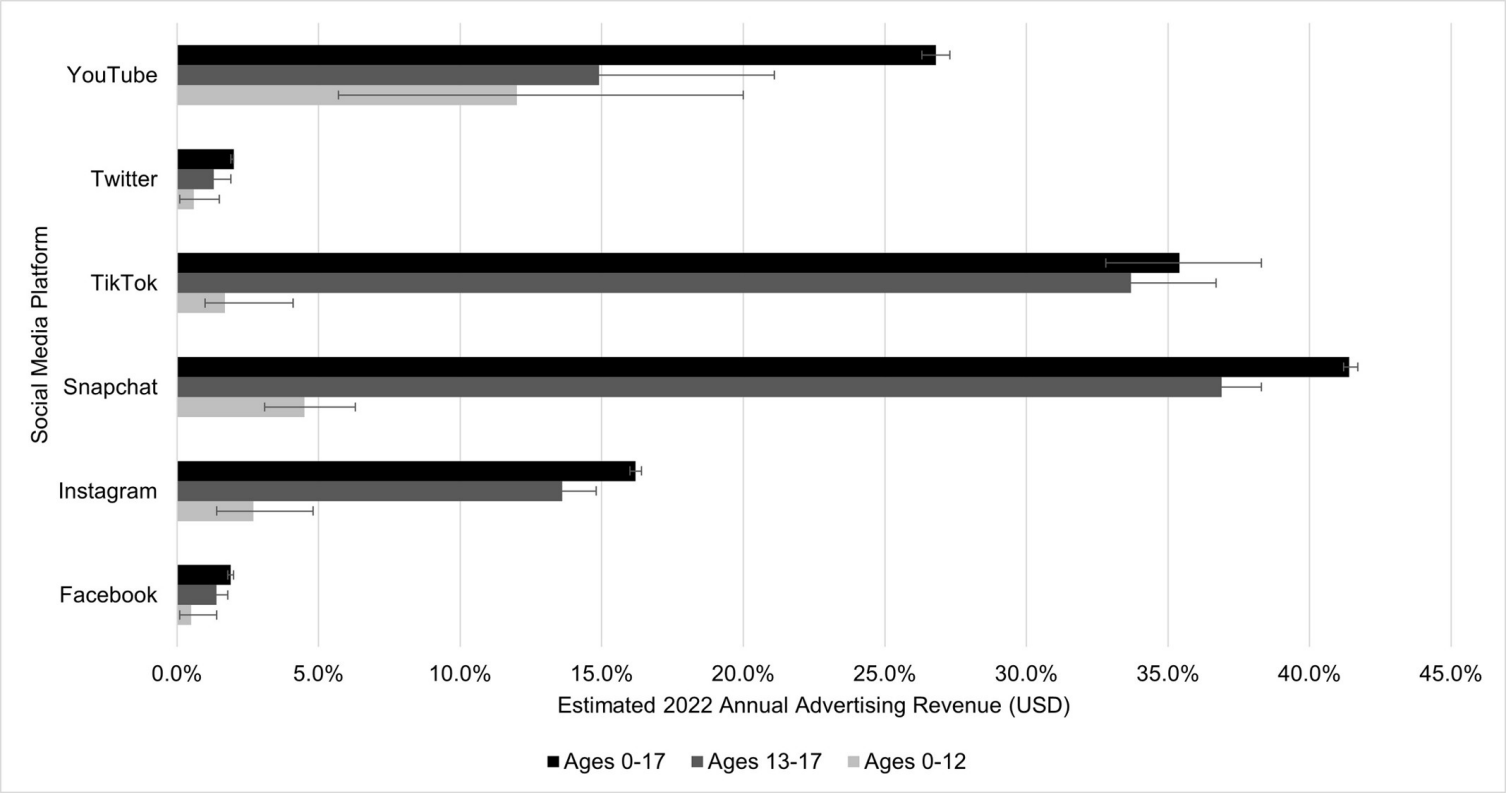
Estimated annual percent of total U.S. advertising revenue from users under 18 years of age across social media platforms, 2022.

The estimated percentage of total advertising revenue derived from users of each platform, separated by age group, are presented in **Fig 2**. On Snapchat, nearly half (41.4%) of overall advertising revenue in 2022 is estimated to be derived from youth ages 0–17 years, followed by 35% on TikTok, 27% on YouTube, and 16% on Instagram. The revenue derived from youth under 18 years is comparatively low for Facebook and Twitter, where the estimated percent of total annual advertising revenue generated from users ages 0–17 years are 1.9% and 2.0%, respectively. This is likely due to the low number of youth users on each of these platforms relative to adult users (see Table 1).

## Discussion

This is the first known study to estimate social media platform-specific advertising revenue from youth. Across the six major social media platforms, annual advertising revenue from children in the U.S. ages 0–12 years is over $2 billion USD and from all youth ages 0–17 years is nearly $11 billion. Approximately 30–40% of the advertising revenue generated from three social media platforms (Snapchat, TikTok, YouTube) is attributable to young people. Government efforts to update protections for children and adolescents have largely failed, facing substantial resistance from industry, and our findings underscore the financial incentive for platforms to oppose government efforts to protect youth.

Given the rise in social media use among youth during the COVID-19 pandemic [17], there has been growing interest in federal and state-level government regulation of platforms’ access to youth. One recent bill, the Kids Online Safety Act (*S*.*1409*, https://www.congress.gov/bill/118th-congress/senate-bill/1409) aims to bolster protections for youth on social media and increase the minimum permissible age of behavioral data collection by platforms, which drives computational advertising practices, to 16 years of age. The Kids Online Safety Act also includes parameters requiring platforms to make data available to researchers, which would vastly improve transparency and improve inquiries into the harms that social media poses to youth. Greater transparency in platform data sharing will allow researchers to obtain reliable estimates for not only advertising revenue, but also user demographics as well as behavioral data relevant to mental health risks associated with social media use.

There were several limitations to our study methods and analysis. We heavily relied upon secondary estimated and projected data, as well as the assumption that youth and adults may see a similar number of advertisements; however, as previously described, social media platforms do not publicly disclose any data on user base ages, nor the advertising revenue generated from them. The reliance on eMarketer presents additional challenges considering the company does not publicly disclose the methods it uses for estimated projected revenue, but we have tried to ameliorate the lack of transparency by providing eMarketer estimates in S2–S4 Tables. To our knowledge, there are no other sources of data that provide the number of child and adolescent users on each platform nor advertising revenue estimates. Our simulation methods offer the most rigorous estimates to date and can be updated as more data are made available.

Future research should investigate the potential health risks of social media-based advertising to youth in the U.S., as has been done in other countries for general media [18], and how influencer-sponsored posts strategically target youth through social media platforms [19]. Researchers and policymakers should also continue to seek alternative sources of data to address the current gaps in publicly available information.

## Conclusions

We undertook this study to inform health researchers and policymakers about how much revenue is generated from youth users and how many children and adolescents actively use the major social media platforms, with a goal to inspire greater data transparency and political will to protect young people online. Our estimates suggest that social media platforms derive considerable profits from youth users, emphasizing the need for greater transparency and regulation of their practices to ameliorate potential harms to youth mental health.

## Supporting information

S1 Table Summary of estimates of social media platform use by age group in the U.S. Estimates for children ages 8–18 were derived from a nationally representative survey conducted by Common Sense Media [1]. Estimates for adults ages 18+ years were derived from a nationally representative survey of 1,502 U.S. adults conducted by Pew Research [14]. *Reported for online videos total. We used estimates of users who report watching ‘every day’.
(DOCX)

S2 Table Summary of estimated total advertising revenue for social media platforms in the U.S., 2022. Estimates of projected annual gross advertising revenue in 2022 for each platform were derived from eMarketer [15], a business marketing research company.
(DOCX)

S3 Table Summary of estimated total users by age group for social media platforms in the U.S., 2022. Estimates of total users (at least once per month) of each platform by age group from eMarketer [15].
(DOCX)

S4 Table Summary of estimated minutes per day for social media platforms among children and adults in the U.S., 2021–2022. Estimates for children were derived from a representative survey conducted by Qustudio in 2021 [16]. Estimates for adults ages 18+ years were derived from eMarketer [15]. *Twitter minutes per day not reported for U.S. children, as it was not among the top 6 apps, the lowest of which (Facebook) was 10 minutes. We therefore fitted the model to a mean of 5 minutes per day.
(DOCX)
