# Supplementary material for: Social media platforms generate billions of dollars in revenue from U.S. youth: Findings from a simulated revenue model
Source: PLoS One. 2023 Dec 27;18(12):e0295337. doi: 10.1371/journal.pone.0295337 (PMC10752512; doi:10.1371/journal.pone.0295337)
Supplement: S1 Table — Estimates for children ages 8–18 were derived from a nationally representative survey conducted by Common Sense Media [1]. Estimates for adults ages 18+ years were derived from a nationally representative survey of 1,502 U.S. adults conducted by Pew Research [14]. *Reported for online videos total. We used estimates of users who report watching ‘every day’. (DOCX) [file pone.0295337.s001.docx]

|  | **Age Groups (years)** | | | | | |
| --- | --- | --- | --- | --- | --- | --- |
| **Platform** | **8-12** | **13-18** | **18-29** | **30-49** | **50-64** | **65+** |
| Facebook | 8% | 30% | 70% | 77% | 73% | 50% |
| Instagram | 10% | 53% | 71% | 48% | 29% | 13% |
| Snapchat | 13% | 49% | 65% | 24% | 12% | 2% |
| TikTok | 64%* | 77%* | 48% | 22% | 14% | 4% |
| Twitter | .. | 16% | 42% | 27% | 18% | 7% |
| YouTube | 64%* | 77%* | 95% | 91% | 83% | 49% |

**S1 Table. Summary of Estimates of Social Media Platform Use by Age Group in the U.S.** Estimates for children ages 8-18 were derived from a nationally representative survey conducted by Common Sense Media [1]. Estimates for adults ages 18+ years were derived from a nationally representative survey of 1,502 U.S. adults conducted by Pew Research [14]. *Reported for online videos total. We used estimates of users who report watching ‘every day’.
