# Supplementary material for: Social media platforms generate billions of dollars in revenue from U.S. youth: Findings from a simulated revenue model
Source: PLoS One. 2023 Dec 27;18(12):e0295337. doi: 10.1371/journal.pone.0295337 (PMC10752512; doi:10.1371/journal.pone.0295337)
Supplement: S2 Table — Estimates of projected annual gross advertising revenue in 2022 for each platform were derived from eMarketer [15], a business marketing research company. (DOCX) [file pone.0295337.s002.docx]

| **Platform** | **Net Ad Revenue (USD)** |
| --- | --- |
| Facebook | $25,750,000,000 |
| Instagram | $29,790,000,000 |
| Snapchat | $2,723,700,000 |
| TikTok | $5,960,000,000 |
| Twitter | $3,010,000,000 |
| YouTube | $8,020,000,000 |

**S2 Table. Summary of estimated total advertising revenue for social media platforms in the U.S., 2022.** Estimates of projected annual gross advertising revenue in 2022 for each platform were derived from eMarketer [15], a business marketing research company.
