# Supplementary material for: Social media platforms generate billions of dollars in revenue from U.S. youth: Findings from a simulated revenue model
Source: PLoS One. 2023 Dec 27;18(12):e0295337. doi: 10.1371/journal.pone.0295337 (PMC10752512; doi:10.1371/journal.pone.0295337)
Supplement: S3 Table — Estimates of total users (at least once per month) of each platform by age group from eMarketer [15]. (DOCX) [file pone.0295337.s003.docx]

| **Platform** | **Age Groups (years)** | | | | | | | |
| --- | --- | --- | --- | --- | --- | --- | --- | --- |
|  | **0-11** | **12-17** | **18-24** | **25-34** | **35-44** | **45-54** | **55-64** | **65+** |
| Facebook | 1,900,000 | 8,000,000 | 16,900,000 | 39,200,000 | 34,100,000 | 28,200,000 | 24,500,000 | 25,500,000 |
| Instagram | 2,400,000 | 14,300,000 | 21,300,000 | 36,800,000 | 24,700,000 | 14,600,000 | 8,400,000 | 5,900,000 |
| Snapchat | 1,600,000 | 16,400,000 | 24,700,000 | 25,100,000 | 11,400,000 | 5,000,000 | 2,700,000 | 1,300,000 |
| TikTok | 2,300,000 | 16,600,000 | 22,500,000 | 23,700,000 | 16,100,000 | 6,300,000 | 4,800,000 | 1,700,000 |
| Twitter | 1,500,000 | 5,500,000 | 10,600,000 | 12,900,000 | 10,500,000 | 7,500,000 | 4,500,000 | 4,300,000 |
| YouTube | 27,800,000 | 22,000,000 | 26,000,000 | 39,400,000 | 34,100,000 | 29,800,000 | 25,200,000 | 26,300,000 |

**S3 Table. Summary of estimated total users by age group for social media platforms in the U.S., 2022.** Estimates of total users (at least once per month) of each platform by age group from eMarketer [15].
