# Supplementary material for: Social media platforms generate billions of dollars in revenue from U.S. youth: Findings from a simulated revenue model
Source: PLoS One. 2023 Dec 27;18(12):e0295337. doi: 10.1371/journal.pone.0295337 (PMC10752512; doi:10.1371/journal.pone.0295337)
Supplement: S4 Table — Estimates for children were derived from a representative survey conducted by Qustudio in 2021 [16]. Estimates for adults ages 18+ years were derived from eMarketer [15]. *Twitter minutes per day not reported for U.S. children, as it was not among the top 6 apps, the lowest of which (Facebook) was 10 minutes. We therefore fitted the model to a mean of 5 minutes per day. (DOCX) [file pone.0295337.s004.docx]

| **Platform** | **Children (minutes)** | **Adults (minutes)** |
| --- | --- | --- |
| Facebook | 10 | 30.1 |
| Instagram | 39 | 30.1 |
| Snapchat | 84 | 30.4 |
| TikTok | 99 | 45.8 |
| Twitter | <10* | 34.8 |
| YouTube | 61 | 45.6 |

**S4 Table. Summary of estimated minutes per day for social media platforms among children and adults in the U.S., 2021-2022.** Estimates for children were derived from a representative survey conducted by Qustudio in 2021 [16]. Estimates for adults ages 18+ years were derived from eMarketer [15]. *Twitter minutes per day not reported for U.S. children, as it was not among the top 6 apps, the lowest of which (Facebook) was 10 minutes. We therefore fitted the model to a mean of 5 minutes per day.
